# Barriers and facilitators to the access to specialized female genital cutting healthcare services: Experiences of Somali and Sudanese women in Norway

**DOI:** 10.1371/journal.pone.0257588

**Published:** 2021-09-17

**Authors:** Mai Mahgoub Ziyada, R. Elise B. Johansen

**Affiliations:** 1 Section for Trauma, Catastrophes and Forced Migration—Adults and Elderly, Norwegian Centre for Violence and Traumatic Stress Studies, Oslo, Norway; 2 Faculty of Medicine, Institute of Health and Society, University of Oslo, Oslo, Norway; Ordu University, TURKEY

## Abstract

**Background:**

Girls and women subjected to female genital cutting (FGC) risk experiencing obstetrical, gynecological, sexual, and psychological health problems. Therefore, Norway has established low-threshold specialized healthcare services where girls and women with FGC-related health problems can directly seek medical attention. Nevertheless, we lack data about access to these services, especially for non-maternity-related purposes. In this article, we explore experiences of seeking medical attention for health problems that are potentially FGC-related, aiming to identify factors that hinder or facilitate access to FGC-specialized services.

**Methods:**

We conducted a qualitative study in three Norwegian cities employing semi-structured repeat interviews with 26 girls and women subjected to FGC, participant observation, and three validation focus group discussions with 17 additional participants. We thematically analyzed the data and approached access as a dynamic process of interactions between individuals and the healthcare system that lasts from an initial perception of need until reception of healthcare appropriate to that need.

**Findings:**

We identified several barriers to healthcare, including 1) uncertainty about FGC as a cause of experienced health problems, 2) unfamiliarity with FGC-specialized services, 3) lack of assessment by general practitioners of FGC as a potential cause of health problems, and 4) negative interactions with healthcare providers. In contrast, factors facilitating healthcare included: 1) receiving information on FGC-related health problems and FGC-specialized services from a non-profit immigrant organization, 2) referral to gynecologists with good knowledge of FGC, and 3) positive interactions with healthcare providers.

**Conclusion:**

Assessing whether FGC is the cause for experienced health problems requires diagnostic competency and should not be left entirely to the patients. We recommend that Norwegian policymakers acknowledge the central role of GPs in the clinical management of patients with FGC-related health problems and provide them with comprehensive training on FGC.

## 1 Introduction

Migrant health and equitable access to healthcare for migrants are gaining importance in many European countries [1,2]. In this article, we explore healthcare-seeking experiences in Norway related to female genital cutting (FGC).

The World Health Organization (WHO) defines FGC, also known as female circumcision and female genital mutilation (FGM), as all procedures that involve the partial or total removal of the external female genitalia or other injuries to the female genital organs for non-medical reasons [3]. The WHO further classifies FGC into four types. The first three increase in severity from the partial or total removal of the clitoris in type I to the sealing of the vulva except for a small hole for passing both urine and menstrual blood in type III (infibulation). On the other hand, the fourth type (IV) includes all other harmful procedures to the female genitalia, including practices such as pricking and piercing.

FGC is prevalent in 30 countries in Africa, Asia, and the Middle East, with over 200 million girls and women estimated to have undergone the practice [4]. Migration from these countries to other parts of the world has resulted in a minority population affected by FGC in Europe [5–8], the USA [9], Australia [10], and Canada [11]. In Europe, over half a million immigrant girls and women had undergone FGC, of which around 17,000 live in Norway [5,7].

FGC exposes affected girls and women to a series of physical and psychological health problems throughout their lives [12–16]. The frequency and severity of these problems vary according to FGC type and specific conditions under the procedure, including hygiene, anesthesia, anatomical knowledge of the circumciser, etc. [17]. Both the clitoris and its surrounding genital tissues have a dense nerve and blood supply [18]; hence injury or damage to these tissues could cause immediate complications such as severe pain, urinary retention, bleeding (hemorrhage), and hemorrhagic shock [3,12]. In the long-term, the injury to either the clitoris, the surrounding tissues, or both could lead to the formation of inelastic scar tissue, keloids, cysts, and neuromas [12,16]. These conditions, together with the obstruction of the vaginal and urethral opening in type III, causes a series of physical health problems, including increased risk of cesarean section, episiotomy, perineal tears, postpartum hemorrhage, stillbirths, urinary- and reproductive- tract infections, prolonged and painful urination, menstrual problems, difficult and painful sexual intercourse, and reduced sexual desire and satisfaction [12–14,19–23]. Furthermore, pain and trauma could lead to psychological health problems, including mental health problems (e.g., irritability, sadness, anger, distress, flashbacks and nightmares, low self-esteem, and relationship problems) and mental health disorders such as depression, anxiety disorders, and post-traumatic stress disorder (PTSD) [14,24–27]. In addition, recent evidence indicates a possible association between stigma and low self-esteem and an increased risk of psychological and sexual health problems among girls and women with FGC living in countries of migration where the majority population has negative attitudes towards FGC [19,28–31].

Consequently, girls and women subjected to FGC might need specialized healthcare such as sexual and psychological counseling, removal of cysts, and deinfibulation [16]. Deinfibulation is a minor surgical procedure whereby healthcare providers release the infibulation seal to expose the vaginal and urethral openings [32–35]. Although healthcare providers mostly perform deinfibulation during pregnancy or childbirth, they can also conduct it for non-obstetrical reasons such as easing the urinary and menstrual flow, facilitating penetrative vaginal intercourse, and reducing sexual pain.

In Norway, specialized healthcare services for FGC-related health problems are primarily deinfibulation and removal of cysts [36,37], available at the specialist healthcare level at all central women’s outpatient departments.

Typically, access to Norwegian specialist healthcare would require a referral from healthcare providers at the primary healthcare level, primarily general practitioners (GPs) [38]. GPs characteristically provide regular, emergency, or acute healthcare services at their medical practices (within office hours) or urgent care centers (after hours). Therefore, all residents are entitled and encouraged to register with a regular GP to ensure both primary care and referrals whenever necessary. In addition, specialists could issue referrals to other specialist healthcare services. Patients commonly pay a subsidized consultation fee when visiting their GP, the urgent care centers, and outpatient healthcare specialists. Co-payments vary between 18 USD per visit for regular GP and 40 USD for outpatient healthcare specialists.

To increase the accessibility of the FGC-specialized services, some hospitals have exempted girls and women with FGC-related health problems from the otherwise required referral and allowed them to contact their women’s outpatient departments directly. Nevertheless, two recent studies [39,40] at two of these outpatient departments identified only 358 girls and women as patients attending FGC-related services during a 10–12 years period. It is noteworthy that these two outpatient departments are located in two large cities (Oslo and Trondheim) that accommodate a high number of immigrants originating from FGC practicing countries [41]. Furthermore, the vast majority of these 358 girls and women did not take direct contact but were referred by other healthcare providers [39,40]. Besides the complaints given as reasons for referral, many of these girls and women were found to have numerous genitourinary and sexual problems that they did not seek help for previously. Therefore, it does not seem that exemption from referral had increased the accessibility of FGC-specialized services for the target group. Unfortunately, we do not know enough about factors that hinder or facilitate their journey while seeking medical attention at the FGC-specialized services, especially for non-maternity-related purposes. We realize that some women prefer deinfibulation during childbirth rather than in the second trimester [40]. We also know that some girls find premarital deinfibulation unacceptable because it contradicted some sexual and cultural norms [42–44]. A very recent study [45] gave new insights on other barriers to help-seeking and access to healthcare that 13 girls and women subjected to FGC had experienced in Norway. Identified barriers included lack of information on both FGC-related health complications and healthcare services, avoidance of disclosing FGC-related health problems to healthcare providers because of shyness and shame, insufficient knowledge of FGC among healthcare providers, and healthcare providers being more concerned about the criminal aspects of FGC than the women’s healthcare needs. Still, we need to know more about the experiences of girls and women who managed to receive medical attention at the FGC-specialized services in Norway: *What motivated these girls and women to seek medical attention*? *Why did they not contact the FGC-specialized services directly*? *Where did they seek medical attention before referral to FGC-specialized services*? *What barriers*, *facilitators*, *or both did they encounter while seeking medical attention for FGC-related health problems*? *How did their experiences differ from those who did not receive medical attention at the FGC-specialized services*?

Understanding the barriers and facilitators encountered by the target group to access FGC-specialized services could guide healthcare providers and health authorities to improve the current services and ensure equitable healthcare for this minority group. This article aims to identify factors that hinder or facilitate access to the FGC-specialized services among Somali and Sudanese subjected to FGC. We explore the experiences of those who did and those who did not receive medical attention at the FGC-specialized services. As most research on access to healthcare has been on maternity-related issues, we limit our focus to our participants’ non-maternity-related healthcare experiences.

### 1.1 Theoretical framework

Access to healthcare is an essential yet ambiguous concept with wide variations in its interpretation and conceptualization in existing literature [46]. Two recent and popular conceptualizations on access to healthcare are Levesque et al. (2013) conceptual framework [46] and Dixon-Wood et al.’s (2005) candidacy theory [47]. Both conceptualizations approach access as a dynamic process of interactions between individuals and healthcare systems, starting with the individuals’ perceptions of a need and culminating with healthcare responding appropriately to this need. Levesque et al. (2013) frame access as the result of the interaction between five characteristics of the population and five characteristics of the healthcare system. The five characteristics of the population include the ability to: perceive healthcare needs, seek healthcare, reach, pay, and engage with healthcare services. Correspondingly, the healthcare system characteristics include approachability, acceptability, availability and accommodation, affordability, and appropriateness of the healthcare services. They suggest that health literacy and beliefs about health and sickness influence the ability to perceive healthcare needs. Hence, providing individuals with proper health knowledge will improve their perceptions of healthcare needs eligible for medical attention and ultimately enhance their access to appropriate healthcare. An underlying assumption is that health problems suitable for medical attention are static, and patients’ limited health literacy hinders their access to proper healthcare. However, Dixon-Wood et al. (2005) challenge this underlying assumption and claim that eligibility for medical attention is negotiated between individuals and healthcare services. They argue that in the same way individuals continually strive to constitute and define what they perceive to be appropriate objects for medical attention, healthcare services are constantly forming and defining the “proper objects” of medical attention. Dixon-Wood et al. (2005) emphasize that the various healthcare services often are organized around an *“ideal user”* with a particular set of competencies and resources required for precisely using the services in a specific manner and for intended health problems.

Hence, for patients to successfully enter and use the healthcare services, their health knowledge and other necessary competencies (usually patterned by age, gender, social class, or ethnicity) need to align with the professional and organizational cultures of the healthcare services. To accommodate the duality of healthcare, Dixon-Wood et al. (2005) propose a concept of candidacy that describes eligibility for medical attention as a dynamic negotiation between individuals and healthcare services. They highlight different stages along the journey for medical attention where such negotiations typically occur. These stages include identification of candidacy, navigation of the healthcare system, claim of candidacy, adjudication of candidacy, and responses to offers of healthcare services. At each stage, candidacy is influenced by a different set of factors. In the first three stages, these factors include individuals’ abilities to: identify health problems that need medical attention, navigate the healthcare services, and claim their candidacy. In the subsequent stages, candidacy is influenced by the permeability of the healthcare services, the healthcare providers’ perceptions of the availability and suitability of resources needed to address the patients’ claims to candidacy, the dynamics of the interactions between patients and healthcare providers, and the acceptability of offered services to the patients.

While we initially used the conceptualizations of both Levesque et al. (2013) and Dixon-Wood et al. (2005) in analyzing the data for this article, as the analysis progressed, we found Dixon-Wood et al. (2005) concept of candidacy to best capture the complexities of our findings. For example, we found that receiving information on FGC-related health problems had helped the participants suspect that their health problems were FGC-related. Nevertheless, they had only accessed the FGC-specialized services when the healthcare providers had authorized these health problems as FGC-related. Thus, while both conceptualizations fitted the first of these two findings, only the candidacy theory remained a good fit for the second. Hence, we chose to use the candidacy theory to inform the organization and interpretation of our findings.

## 2 Methods

This article draws on data from an explorative qualitative study examining the perceptions and experiences of Somali and Sudanese participants in Norway on healthcare needs and healthcare services related to FGC. We conducted this study in 2016–2018 utilizing semi-structured repeat interviews with 26 participants, participant observation, and three validation focus group discussions (FGD) with 17 additional participants. In this article, we explore the experiences with seeking medical attention for potential FGC-related health problems to identify factors that hinder or facilitate access to non-maternity-related FGC-specialized services.

### 2.1 Setting, recruitment, ethics, and participants

Somali and Sudanese are among the largest FGC-affected groups in Norway [7]. Therefore, we recruited the study participants from three Norwegian cities (Oslo, Drammen, and Trondheim) that accommodate large numbers of girls and women with origins from both countries.

We recruited the study participants through different starting points using purposeful sampling, as described in Patten (2015) [48], to ensure a varied sample in terms of age, marital status, education, type of FGC, and length of stay in Norway. In addition, we purposefully recruited participants in the age group 16–25 years as young women have been underrepresented in previous studies.

We informed all potential participants about the study’s aim and ethical and practical issues relevant to them if they agreed to participate. In addition, we oriented them about the voluntary nature of participation and our plan for data storage and management. To include potential participants who might have difficulties giving written consent, we asked the ethical committee to permit oral consent. Permission was granted. Therefore, per the ethical clearance for this study, both verbal and written consent from potential participants who had professed to understand the provided information and had agreed to participate are accepted as informed consent. Hence, we asked all who consented to join for oral or written consent, including those aged 16–18 years. The Norwegian Health Research Act allows minors to give independent consent from 16 years, except for clinical drug trials or other research projects involving bodily interventions. Although the ethical committee did not stipulate any form of documentation for oral consent, we voice-recorded the verbal consent of all participants who had granted recording. We stopped recruitment once we ceased to observe new information. Table 1 outline the general characteristics of the participants.

**Table 1:** Overview of participants’ characteristics.

Characteristic	Participants	
	Semi-structured repeat interviews (n = 26)	Focus group discussions (n = 17)
Background		
Somalia	11	11
Sudan	15	6
Age (years)		
16–21	9	1
22–27	8	5
28–33	1	4
34–39	2	4
40–45	3	2
≥ 46	3	1
Marital status		
Married	10	7
Divorced	5	4
Single	11	6
Education		
≤ Middle school	2	1
High school	9	11
College	10	4
Graduate school	5	1
Type of FGC		
Type I	3	0
Type II	4	5
Type III	19	12
Length of stay		
< 1 year	1	0
1–5 years	6	4
6–10 years	5	5
>10 years	14	8

This study was initially approved in 2016 by the Norwegian Social Science Data Services. In 2017, the Norwegian Regional Committee for Medical and Health Research Ethics approved the study as a sub-study (Ph.D.) within a large project. Consistent with the ethical clearance, we did not pay the participants for their participation but compensated many with a gift card (the equivalent of 30 USD) for transport, wages lost, or both.

### 2.2 Interviews, participant observation, and focus group discussions

The first author conducted all semi-structured repeat interviews, participant observations, and two FGDs, while the second author conducted the third FGD. We performed all interviews in participant-selected locations, such as cafés and homes of either the first author or the participants. In contrast, we carried out the FGDs on the premises of local immigrant organizations. The first author is a native Arabic speaker, fluent in English, and has moderate competence in Norwegian. Hence, she conducted the interviews and FGD with the Sudanese participants in Arabic and the Somali participants in English, Arabic, or a mixture of English and Norwegian.

The first author conducted 61 semi-structured interviews with 26 participants, of whom she interviewed 17 twice and 9 three times. All 26 participants we invited had agreed to a second interview, whereas only nine out of 10 invited participants had agreed to a third interview. The first-time interviews aimed to build trust and encourage the participants to narrate experiences related to their FGC. In the second time interviews, the participants were encouraged to elaborate further on their FGC experiences by revisiting the narrations from the first interviews, with the first author probing for confirmations, corrections, and elaborations. Using a semi-structured interview guide [44], the first author asked the participants to narrate what they could remember and wanted to share concerning their circumcision experiences, experiences of health problems they attributed to the circumcision, and how they managed these health problems and why. The first-time interviews lasted between 30–90 minutes and the second 60–180 minutes. In both interviews, the participants’ narrations went mostly uninterrupted and with minor probing. Although the first-time interviews were unrecorded, the first author had tried to minimize any recall bias by recording her recollections and interpretations immediately after each interview. However, all of the second-and third-time interviews were audio-recorded.

The purpose of the third time interview was to member-check our preliminary analysis. Here, we presented our initial interpretations of emerging patterns and themes to each of the nine participants for further discussion and clarification. These interviews lasted between 60–90 minutes. Finally, we sought to validate our preliminary interpretations of emerging patterns from the three rounds of interviews through three validation FGDs with 17 Somali and Sudanese participants. We presented our overall findings for discussion and probed for confirmations, disagreements, modifications, and elaborations. We recorded our notes from the FGDs and saved them as audio files.

Moreover, the first author participated in over 20 seminars wherein girls and women subjected to FGC shared their experiences with FGC and the healthcare system in Norway. Following each seminar, the first author recorded her observation as audio field notes and used them to inform the semi-structured interview guide and the data analysis.

### 2.3 Analysis

The first author analyzed the data in close discussion with the second author. We entered all audio files into NVivo 12 and separated each data source (i.e., interviews, FGD, and participant observations). We identified recurrent themes and patterns in all interviews following the thematic analysis approach described by Braun and Clarke [49]. The only difference is that we did not transcribe the audio files first. Instead, the first author listened to each interview twice in its entirety before assigning codes to relevant audio segments. She kept adding to- and refining- initial codes while coding all of the participants’ two first interviews. She then used insights from all third interviews and discussions with the second author to refine the codebook. Next, we sorted the codes into potential themes and collated all corresponding data extracts under these initial themes. We reviewed and refined these themes repeatedly until we developed well-identified themes and sub-themes. The first author transcribed and translated the collated audio segments directly to English whenever necessary. Notes from the FGDs and participant observation added further insights and weight to the findings. Using the candidacy theory [47] as an additional lens, we refined the themes and sub-themes addressing our objective and combined them into a coherent description. To minimize the risk of recognizing the participants, we have used pseudonyms and slightly altered- or kept- the participants’ characteristics vague and ambiguous. Finally, we followed the checklist of consolidated criteria for reporting qualitative research (COREQ) [50] to facilitate peer assessment of the article’s trustworthiness criteria (i.e., credibility, transferability, dependability, and confirmability).

### 2.4 Reflexivity

The first author probably gained the trust of both the Sudanese and Somali participants by striking a good balance between her roles as insider and outsider, as described by Kusow [51]. As a Sudanese female doctor born and raised in Sudan, we expected both Somali and Sudanese participants to perceive her as someone who shared experiences and understanding of cultural subtleties and knowledge of FGC and thus find it easy to communicate their perceptions and experiences. Still, we were concerned that by sharing the same background as the researcher, some Sudanese participants could be afraid of gossip and judgment and consequently reluctant to share opinions or experiences that deviated from the community’s socio-cultural norm. Therefore, the first author repeatedly explained confidentiality issues, de-identification, and possible repercussions of any deviation from these ethical principles. We also think that the first author’s departure from the local Sudanese norms, demonstrated by her marriage to a Westerner, led some participants to regard her as partly an outsider. Furthermore, the first author’s medical background seemed to have further facilitated trust and openness. Some women provided it as a reason for finding it easy to share information they had not previously shared.

## 3 Findings

We explored the participants’ experiences with seeking healthcare for potential FGC-related health problems along a spectrum that starts with the initial perception of health problems and culminates with the reception of appropriate healthcare. We found that most participants had experienced a series of health problems that could have been related to FGC at one or more points across their lifespan. During childhood, the most common complaints described by participants were burning micturition, recurrent itching, and sores on their genitals. Post-puberty, the most common complaints were mental and sexual health problems, severe menstrual pain, and recurrent painful vulvar lumps.

Nevertheless, we identified different factors (see Table 2 for a summary of these factors) that influenced whether these participants had received appropriate healthcare for these problems.

**Table 2:** Barriers and facilitators to the access to FGC-specialized services in Norway.

Theme	Barriers	Facilitators
**Identification of candidacy**	• Uncertainty regarding whether genitourinary problems were FGC-related: ○ Conflicting opinions from healthcare providers, and ○ Conflicting views from people of trust.	• Experiencing health problems that: ○ Caused severe pain, ○ Persisted despite attempts of self-management, ○ Were lumps that raised the participants’ concern over malignancy, and ○ Interfered with their ability to perform expected duties and roles. • Receiving information on FGC-related health problems, and • Linking experienced sexual and mental health problems to FGC: ○ Consistent endorsement of the link between sexual and mental health problems and FGC by healthcare providers and trusted others.
**Navigating the healthcare system**	• Language barriers and limited knowledge of the Norwegian healthcare system as newcomers, • Limited knowledge of FGC-specialized services: ○ Not accessing available information on official websites, and ○ Lack of information on sexual and psychological counselors familiar with FGC. • Practical issues with appointments: ○ Language, ○ Invalid numbers or switchboard operators not familiar with FGC-specialized services, and ○ Co-payment fees being high for students.	• Having a social network with good language competence and knowledge of the Norwegian system, • Residing in municipalities that assisted during settling, and • Receiving information on FGC-specialized services from local immigrant organizations.
**Permeability and adjudication of candidacy**	• Seeking healthcare at regular GPs and not FGC-specialized services, • FGC not assessed as a possible cause of health problems: ○ Not disclosing FGC status for shame and fear of judgment, ○ GPs not asking about FGC/ not linking health issues to FGC, and ○ Complaints of severe menstrual pain not taken seriously. • Long gynecologists’ waiting lists, and • Feeling judged and disrespected.	• Informing the GPs that they suspected FGC to cause the health problem, and • Being referred to gynecologists with good knowledge of FGC.
**Response to offers and satisfaction with FGC-specialized services**	• Sexual norms rendering deinfibulation unacceptable, • The healthcare providers did not address needs for: ○ Respect, ○ Proper consultations and enough time to deliberate over decisions, ○ Assurances on: ◾ The aesthetic effect of deinfibulation on the vulva, ◾ Making deinfibulation a less traumatic and painful experience, and ◾ Involving them in decisions about pain management. • Psychosexual counseling was not part of the offered FGC-specialized services.	• Sexual norms rendering deinfibulation acceptable/desirable, • The healthcare providers accommodated needs for: ○ Respect. ○ Proper consultations and enough time to deliberate over decisions, ○ Assurances on: ◾ The aesthetic effect of deinfibulation on the vulva, ◾ Making deinfibulation a less traumatic and painful experience, and ◾ Involving them in decisions about pain management, • Experiencing a positive impact of deinfibulation on health problems.

### 3.1 Identification of candidacy

We identified two barriers that have deterred the participants from seeking medical attention at the FGC-specialized services. Firstly, we found that despite the magnitude of experienced health problems, not all participants had identified these problems, and consequently neither themselves, as candidates for medical attention. Typically, the participants had first tried home remedies before considering other options. For example, the participants took over-the-counter painkillers, drank warm herbal tea, and placed hot bottles on their bellies for severe menstrual pain. They had also carefully attended vulvar lumps and gently squeezed out the pus until it was empty and no longer painful. Only when they could not tolerate the pain, could no longer self-manage these health problems, or were concerned that the vulvar lump could be a cancerous tumor had they consulted others or sought medical attention. One such participant was Aaraan, a 23 years old Somali woman who had suffered for several years from repeated vulvar lumps that the gynecologist eventually diagnosed as benign cysts. Aaraan described her reason for finally seeking medical attention after many years of self-management as follow:

*“I remember from my late childhood … until I was twenty, I always had cysts [forming] down there … I remember that sometimes I will open them [the cysts] by myself … to take out what is inside. But, this last time, I could not take it out [the content of the cyst] because it was excruciating… it [the cyst] was closed completely. Previously, they [the cysts] used to have little openings … on the top … there was also a … a crust? But, the last cyst, I could not open that one. So I just left it. I avoided touching and was very careful when I washed my down parts. Then, I went to the gynecologist to know what it could be. I was terrified that I have cancer or something similar.”*

Similarly, not all participants who had experienced mental and sexual health problems perceived themselves to need medical attention. Typically, participants were motivated to seek medical attention only if they perceived that the mental and sexual health problems harmed their intimate relationship, other significant aspects of their lives, or both. More details on the interrelationship between different sets of perceived sexual roles and duties and the participants’ decisions on whether or not to seek medical attention for potential FGC-related health problems are provided in a separate article [44].

Secondly, we found that most participants were uncertain whether their genitourinary problems were related to FGC and thus did not identify themselves as candidates for FGC-specialized services. Most participants had received information on FGC-related health problems at seminars. This information, especially pictures of keloids and cysts, had made them aware of a possible link between their health problems and FGC. However, they had encountered conflicting opinions on some FGC-related genitourinary health problems, particularly menstrual pain, from healthcare providers in Norway and home countries. While some healthcare providers had attributed severe menstrual pain to FGC, others had disagreed and instead attributed such pain to uterine muscle contractions. The participants also claimed to have received equally conflicting messages regarding these health problems from trusted peers. Such contradicting opinions left most participants uncertain whether their menstrual pain and other health problems such as vulvar lumps and urinary infections were related to FGC and thus whether they were candidates for FGC-specialized services.

Only after deinfibulation and experiencing a remarkable improvement in their health problems, some participants had become sure that these health problems had been FGC-related. For example, the before- and after- deinfibulation experiences of menstrual pain had helped them differentiate between “normal” menstrual pain and menstrual pain in “tightly” infibulated girls and women. They explained that pre-deinfibulation, the opening was insufficient for the menstrual blood to flow freely, thus allowing clots to form, accumulate, and add a new dimension to pain in the form of extreme pressure. The following quote from Hadiya, a Sudanese participant in her early-40s, depicts what the participants understood as FGC-related menstrual pain:

*“Initially, I did not relate my menstrual pain to circumcision, but then I felt this immediate difference after deinfibulation. You see, I still get menstrual pain, but it is different. The heaviness is gone. Now, I feel the pain in my belly, in the area around the bladder and uterus. Before, I also had severe pain way down, around the [vaginal] opening… a pressure pain … it was as if you have a three or four kilos weight of something pressing on you. It was the blood not finding a way to come out. It was as if you are giving birth! Severe pressure and pain, and then you suddenly feel a huge clot passing out. You feel this instant relief but then feel the pressure building up again! I do not want to be gross, but I will tell you anyway, the pressure would sometimes be so intolerable that I would insert my fingers and pull the clots out!”*

Similarly, once the participants noticed that their recurring experiences of vulvar itching and burning micturition had stopped after deinfibulation, they became sure these problems were FGC-related. For example, Khalda, a Sudanese participant in her 40s, described her long experience with itching and burning micturition and explained that it was only after deinfibulation that she knew it was FGC-related:

*“[…] since I was a child, I had this problem … repeated itchiness down there. I would scratch and scratch until it bleeds. I could not help myself. So when I go to the bathroom, peeing would be very painful. It was like adding salt to an open wound! So, I would try to hold the urine as long as I could. […] since the surgery [deinfibulation], it is over ten years now … during all this time, I haven’t experienced these problems not even once! So, I know for sure they were related to the circumcision.”*

Some urinary problems, such as delayed- and slow- relief, were mostly normalized and not recognized as problems until after deinfibulation. For example, the following quote from Omayma, a Sudanese participant in her mid-20s, was typical:

*“I only realized that I had problems when I felt a difference in everything after deinfibulation. Previously, whenever I was peeing, I had to wait for some time and squeeze hard to get relieved, but now I get immediate relief.”*

In contrast to genitourinary problems, certainty about the link between FGC and sexual and mental health problems had helped the participants identify their candidacy to FGC-specialized services. Participants claimed that their own experiences, the interpretations of healthcare providers, and trusted peers had consistently endorsed the information they had received in seminars linking mental and sexual health problems such as panic attacks and painful sexual intercourse to FGC. They were, therefore, sure that these health problems were FGC-related. Many participants reported experiencing panic attacks in situations that reminded them of the powerlessness and pain they had felt during their circumcision. They explained that certain smells and activities (e.g., disinfectants and the mere act of lying down during childbirth or examination at the gynecologist) would transfer them to when they were held tightly by one or more women as little girls while another cut their private parts. Some participants also professed to have experienced panic attacks during sexual intercourse. Several participants, mainly in the age group 16–25 years, expressed mixed feelings of anger, deep sadness, and shame over being circumcised, as they believed circumcision has rendered them “mutilated” and “unattractive.”

Many sexually active participants described their sexual debuts as painful and enduring experiences where full vaginal penetration had only been possible after repeated agonizing attempts over periods varying from several days to several months. A few of these participants claimed to continue experiencing severe pain during sexual intercourse even years later. They also claimed that their enduring experiences of sexual debut had left them continuously struggling with feelings of shame and self-doubt. One of these participants was Ilham, a 27 years old Sudanese participant, who eloquently narrated her experience of sexual debut and its lasting effect:

*“It was like a nightmare! The wedding party was over, and we went to the hotel. And there we were, the two of us alone. Like any other bride, I was shy yet excited. It started well, but suddenly in the middle of it all, he stopped. He got up and got dressed. I was confused! He sat next to me and covered me with the bedsheet. He then asked me: ‘you are infibulated, right? Why didn’t you tell me? I never wanted to marry a girl that was circumcised, let alone infibulated! Still, if I knew you were infibulated, we could have arranged for it to be opened’! I was mortified. I wished the ground would open and swallow me up! I told him it wasn’t too late; he could still divorce me and find someone not circumcised to marry. He hugged me and reassured me that was not what he meant. He said he loved me and hence didn’t want to cause me any pain, and that would have been impossible with infibulation. So, if I had told him beforehand, he could have arranged for it [infibulation] to be opened. I eventually calmed down and agreed to go and have it opened by a doctor. But, you know what? Something inside me broke that night! I feel disgusted with myself! He tries to reassure me and tell me that now I am normal down there, but I won’t let him look. I always turn the light off when we make love.”*

Furthermore, many participants said they were either not getting much sexual pleasure or could not reach sexual climax. The professed reduced sexual pleasure left some participants feeling dissatisfied and frustrated and had eventually led to marital problems. They explained that their sexual dissatisfaction, often combined with pain and triggered memories of their circumcisions, had reduced their sexual desire and led them to avoid intimacy with their partners. Consequently, their partners accused them of being “cold” and “frigid,” an accusation that made them feel hurt and anxious.

### 3.2 Navigating the healthcare system

The first barrier that the participants encountered while navigating the healthcare system in Norway was their limited knowledge of the system and the Norwegian language. Many participants explained that before migrating to Norway, the healthcare systems they were familiar with were quite different from the Norwegian system, especially GPs as gatekeepers to specialized services. Therefore, several of these participants depended on their social network to get familiar with the Norwegian healthcare system during the first years after migration. They discussed their health problems with people they trusted and asked where and how to seek help. Consequently, their knowledge about the healthcare system varied according to the knowledge levels among their social network. The participants, whose social network was knowledgeable, were quickly acquainted with the Norwegian healthcare system and had promptly registered with a GP. A few participants had also received considerable help from their municipalities upon settling. In contrast, participants with a less knowledgeable social network took a long time getting familiar with the system and registering with a GP. As a result, these participants sought healthcare primarily at urgent care centers until they finally enrolled with a GP.

The second barrier was their limited knowledge of FGC-specialized services. Even though several official Norwegian websites provide information on FGC-related services in various languages, only two participants had searched for and found relevant information on these sites. Instead, the primary source of health information for both the participants and their social networks was seminars arranged by non-profit immigrant organizations. Still, the participants were frustrated that they had received information on FGC-related health problems but not FGC-specialized services. For some, getting information on health problems but not healthcare had left them angry, sad, and hopeless. For example, *Reem*, a 22 years old Sudanese girl, described her feelings during- and after- such FGC seminars as follow:

*“[…] when she mentioned menstrual pain … I was so angry! I was so angry with my mother for doing this to me! I suffer every month! I miss school and lie in bed for three days. I am in such pain I cannot tolerate being touched. I was already suspecting it was because of the pharaonic circumcision […]. Then, they told us about deinfibulation, after a year, I think, and I was angry with that woman, the one who gave the seminar on health complications. Why did she not tell us that we could get help? Why did she tell us about all those problems and just left us feeling miserable?”*

Nevertheless, the participants were happy that non-profit immigrant organizations had recently started providing information on FGC-specialized services for non-maternity-related health problems. The participants mostly received information on FGC-specialized services from seminars arranged by such organizations, either directly or through their social network. Still, the participants complained over lacking information on sexual and psychological counselors familiar with FGC, especially after knowing psychological and sexual counseling was not part of the offered FGC-specialized services. Several participants claimed that they did not seek professional counseling because of not knowing where to find FGC-competent counselors.

The third barrier participants had faced while navigating the healthcare system was practical issues concerning appointments with either the GP or the FGC-specialized services. Several participants experienced that they needed better command of the Norwegian language to book appointments and adequately communicate their needs for medical attention. They had also found the provided contact numbers for the FGC-specialized services were either invalid or belonged to hospitals’ switchboards where the operators were not familiar with the services. Finally, a few participants found the co-payment fees for FGC-related consultations high for students and the less affluent. Hence, they suggested waiving such fees to encourage these two groups to seek medical attention.

### 3.3 Permeability, claims, and adjudications of candidacy

Limited knowledge of FGC-specialized services and uncertainty of whether some gynecological health problems were FGC-related had deterred participants from seeking medical attention at the FGC-specialized services. Instead, many participants had sought medical attention for potential FGC-related health problems with their regular GPs. Nevertheless, participants encountered three main barriers in their encounters with the GPs that had hampered a proper assessment of whether their health problems were FGC-related and subsequently prevented or delayed their referral to FGC-Specialized services. The first barrier was feelings of shame and embarrassment over undergoing FGC, and fear of being judged had discouraged participants from disclosing their FGC status to their GPs. Consequently, the participants had either reluctantly revealed their FGC status while relaying their potential FGC-related health problems or withheld it but expected the GPs to ask them about their FGC status if relevant. The second barrier was that when the GPs did not ask them about their FGC status or indicated a link between their health problems and FGC, participants concluded that the GPs lacked knowledge on FGC or their health problems were not FGC-related. The following quote from a Somali participant in her late teens illustrates some of these experiences:

*“After I told her [the GP] about my problem [itching, sores, and recurrent lumps in the vulva], I waited to see if she would ask me about circumcision … everyone knows that Somalis are circumcised! So, when she did not ask me about circumcision, I knew that it [the health problem] has nothing to do with circumcision.”*

The GPs had only adjudicated candidacy to FGC-specialized services for participants who proactively claimed their candidacy and told their GP that they suspected infibulation as a cause of their sexual health problems. These participants had typically linked their health problems to FGC but had not known about either the FGC-specialized services or the exemption from referral. Still, the GPs had referred most participants with sexual health complaints and vulvar lumps to outpatient gynecologists at their private practices or the hospitals’ outpatient departments.

The third and last barrier participants encountered in their meetings with GPs mainly concerned severe menstrual pain. These participants claimed that their regular GPs did not take their complaints of severe menstrual pain seriously. Instead, the GPs had only prescribed contraceptive pills, advised them to use over-the-counter painkillers, and had refused to issue more effective painkillers or sick leave. Subsequently, when severe menstrual pain persisted even after months of using the prescribed contraceptive pills and over-the-counter painkillers, these participants had decided to seek help at the urgent care centers. There, they claimed to have received adequate pain management. Also, the doctors at the urgent care centers had linked the severe menstrual pain in two participants to FGC, herewith adjudicating their candidacy to FGC-specialized services and referring them for deinfibulation. The participants thought that this difference in the medical assessment of their menstrual pain was because advanced bookings had prevented their regular GPs from observing the pain intensity and consequently grasping their suffering. Contrastingly, the doctors at the urgent care centers could comprehend their struggle since they could drop in whenever they were in acute pain.

Referral to outpatient gynecologists had also resulted in the adjudication of candidacy. The participants were mostly satisfied with the gynecologists’ knowledge of FGC and their ability to assess whether their health problems were FGC-related before referring them to the FGC-specialized services. Nevertheless, the participants had to overcome also two barriers here. The first was the long waiting list for appointments that left them suffering for several months before their health problems were eventually assessed and diagnosed. The second was negative encounters with the gynecologists. Several participants recalled feeling judged, humiliated, and disrespected by gynecologists who had displayed their shock and disgust and used an aggressive tone to ask them never to subject their daughters to such “inhuman” tradition while examining their genitalia. Following such experiences, participants had avoided undergoing vaginal examinations and disclosing their FGC status to other healthcare providers for a long time. The following quote from Najwa, a Sudanese participant in her early-20s, depicts an early experience with a female gynecologist:

*“She started to examine me and made this face … as if she was disgusted. I told her, ‘by the way, I did not do this to myself. I did not even have a choice.’ She then said, ‘I am sorry, but this is very terrible. I hope you would not do this to your daughters in the future.’ I almost answered back that I will!”*

After such encounters, Najwa and a few other participants had refused further referrals until their GPs ensured that the referrals were to gynecologists with vast experience with FGC.

### 3.4 Responses to offers and satisfaction with FGC-specialized services

At the FGC-specialized services, almost all participants had received offers for deinfibulation, sometimes also removal of cysts, to address their various genitourinary and sexual problems. The participants’ responses to these offers were influenced primarily by their perceived sexual norms and secondarily by the dynamics of their interactions with the healthcare providers. Typically, cultural norms related to accepted sexual behavior had determined the acceptability of deinfibulation for the participants and their final decisions to accept or decline the offers. As we have discussed the influence of sexual norms on the acceptability of the various FGC-specialized healthcare offers in a separate article [44], we will only present our findings on the dynamics between the participants and the FGC-specialized healthcare providers.

We found that the interactions’ dynamics had influenced some participants to decline deinfibulation though they found the procedure acceptable/desirable. Also, the interaction dynamics with the healthcare providers had significantly influenced the participants’ satisfaction with the FGC-specialized services.

Overall, the participants were afraid that the healthcare providers could judge them and that deinfibulation could be as painful and traumatic as their initial experiences of circumcision, lead to painful urination, and leave their vulva wide open like a “gaping hole.” Consequently, they were satisfied and had readily accepted offers of deinfibulation when the healthcare providers had addressed each of these concerns and helped them overcome their fear, and vice versa.

Firstly, participants were concerned that the healthcare providers would not meet them with respect. They were sensitive to having several people inspecting their vulva and characterized such experiences as humiliating and disrespectful. The following quote from Sahra, a Somali girl in her early-20s, illustrates the participants’ reactions to such experience:

*“I would never forget how small I felt at the time. [The gynecologist] had students in the room. She did not ask me if it was okay. I never agreed to have them staring at my private parts! It was a very uncomfortable situation. I felt violated [and] disrespected.”*

In contrast, participants appreciated and highlighted occasions when the gynecologist had asked medical students to leave the room as positive examples of compassion and respect. Najwa was particularly appreciative of the positive dynamics since she had a previous unfortunate experience with one gynecologist:

*“This gynecologist was very good … really considerate. She had students in the room [but] she understood without me saying anything that would be problematic for me. She did not even ask me if I would mind having [the students] there. [Instead,] she politely asked them to step out for a bit. I appreciated that [as]I felt well respected. She gained my trust 100% by doing just that.”*

Secondly, participants wanted proper consultations where the healthcare providers had assessed their health problems, explained why deinfibulation was offered, and gave them enough time to consider these offers. Several participants had repeatedly declined offers of deinfibulation from healthcare providers, assuming it was a routine offer for all infibulated girls and women. Only when the gynecologists had explained how deinfibulation would alleviate their health problems had they considered and accepted the offer. Participants also appreciated being offered more than one consultation to discuss their concerns. Reversibly, the participants were dissatisfied with the FGC-specialized services at one particular women’s outpatient department and claimed they were misinformed about their appointments and pressured to undergo deinfibulation. They described their astonishment to find that their expected consultations were instead appointments for deinfibulation. They were also frustrated over what they described as the “take-it or leave-it” attitude the gynecologists seemed to have adopted. They felt pressured to decide whether to undergo deinfibulation or not at their first appointment. In the following quote, a Somali participant in her late-20s describes her frustration with such attitude:

*“She [the gynecologist] had never told me that I had to have surgery … just a consultation. Also, the letter I got said consultation and not surgery […]. So, when I went to [the FGC-specialize services], I thought the gynecologist would talk to me about my problem … what are my options… a regular consultation! But, she was ready to do a surgery [deinfibulation and cyst removal]; she was not polite or kind. I told her I was not ready for surgery and that I came for a consultation. I knew a lot about the Norwegian health system, I am a nurse, so I knew a lot about patients’ rights, that the patients should receive prior information. [That] there must be a consultation first! I could have had cancer as far as I knew [since] until that point, I did not know what [the cyst] was. So, when she told me that she was ready to do the surgery, I told her I was not ready and came for a consultation and was not prepared to have surgery. She said to me, ‘you have to go through with the surgery now. Otherwise, you have to go home’… So, I told her, ‘okay, then I want to go home.’ So, I left and went home.”*

Still, a few participants appreciated that the gynecologists had adopted a prompt and matter-of-fact attitude and perceived it as a positive display of professionalism that had helped them see deinfibulation as a simple procedure. They had also appreciated not getting time to dwell on their fears.

Finally, participants wanted the healthcare providers to assure them that their vulva would not be “wide and ugly” after deinfibulation and try to make deinfibulation a less traumatic and painful experience and involve them in decisions about pain management. Participants who recalled that the healthcare providers had discussed pain management during- and after-deinfibulation and presented various pictures of vulva before- and after- deinfibulation had praised these healthcare providers for helping them overcome their fears. They recounted these consultations as positive experiences and the healthcare providers as competent, kind, and compassionate. Many participants also singled out one particular midwife who originated from an infibulation-practicing country and used to work at one of the FGC-specialized services as exceptionally competent, compassionate, and helpful. Oppositely, participants who had not experienced that the healthcare providers had addressed these concerns had felt angry and frustrated that they could have spared unnecessary anxiety and wasted time. The following quote from Maryan, a Somali woman in her early-30s, was typical:

*“After going through with the surgery, I found it was not bad as I heard or expected. I think [the gynecologist] could have given me better information about the surgery, but she had many things going on at the same time … many women waiting for their surgeries. Then there were the students and the interpreters. She did not have the time to give me a good explanation about the surgery. She was also not, umm, kind? Listening to her, I thought it would be as a big deal as giving birth! However, it was nothing like that. She could have told me how I would look or feel after the surgery. I was so afraid that I would look like I gave birth to eight kids! I did not want that.”*

Terrified that deinfibulation could be as traumatic as their initial FGC experiences, several participants felt frustrated and powerless when the outpatient gynecologists readily dismissed their requests for general anesthesia. They recalled that they could not stop their bodies from shaking and had even fainted during the procedure. In hindsight, they did not consider general anesthesia necessary, but they wished that the outpatient gynecologists had been compassionate and spent some time addressing their fears. The following quote demonstrates how one of these participants, a Sudanese woman in her early-30s, described such experience:

*“Just the thought of someone cutting me down there again sent me to full panic. I needed it done, but I did not want to be awake. So, I asked [the gynecologist at the FGC-specialized services] to knock me out. I told her how I felt and that the gynecologist who referred me there assured me that I did not need to be awake, but she said that was impossible. She gave me ten minutes to make up my mind [as] she had other patients waiting. So, I had ten minutes to decide whether to do it with local anesthesia or go home. I had spent a long time ensuring I had this day free from work, so I finally agreed. I then asked her to give me strong painkillers at least, but again she refused! She said it was not necessary. She was [acting] cold, and as a matter of fact, she was not kind at all … I do not think I felt any pain, but I was shaking and sweating the whole time.”*

Many participants had also wished that counseling was part of the FGC-related offered services to better deal with the low self-esteem, flashbacks, and panic attacks that many struggled to overcome. Nevertheless, such negative experiences with the operating gynecologists did not affect the participants’ satisfaction with the effect of the services they had received. All participants who had undergone deinfibulation, removal of cysts, or both stated that these procedures had considerably helped their health problems. They had noticed immediate improvement regarding menstrual pain, painful sexual intercourse, and reduced sexual pleasure. A few participants attributed the improvement in sexual pleasure to their clitorises being found intact under the infibulation seal. Still, participants whose clitorises were said not to be intact also claimed a noticeable improvement in sexual pleasure and eventually also in sexual desire. Several participants had also noticed that painful urination and recurrent itching, sores, and painful lumps in their vulva had stopped after deinfibulation. Many were also positively surprised by the improved urinary flow.

## 4 Discussion

We explored the experiences of 26 Somali and Sudanese girls and women subjected to FGC to identify factors that had hindered or facilitated their access to non-maternity-related FGC-specialized services in Norway. We approached access as a dynamic process of interactions between individuals and the healthcare system that lasts from an initial perception of need until reception of healthcare appropriate to that need. As a result, we identified several hindering and facilitating factors at four different stages of their healthcare-seeking journeys (Table 2). To lift our findings and enhance their transferability [52] to other contexts, we have initially used both Levesque et al. (2013) conceptual framework [46] and Dixon-Wood et al.’s (2005) candidacy theory [47] to analyze the data for this article. However, as the analysis progressed, only the candidacy theory remained a good fit for the findings. Thus, we have organized and interpreted our results only in line with the candidacy theory.

We found that in the first stage of their healthcare-seeking journey, the participants’ health problems they identified as candidates for medical attention were: lumps and other genitourinary, sexual, and mental health problems that persisted, interfered with their ability to perform expected duties and roles, or caused severe pain. Dissimilar to participants in a former study [45], most of our participants had received information on FGC-related problems and thus suspected their health problems could be FGC-related. A possible explanation for this difference could be that other immigrant groups included in the before mentioned study are less targeted with such information than Somali and Sudanese [53], who were the only groups we included in our study. Still, conflicting messages from healthcare providers and trusted peers had left our participants unsure whether their genitourinary health problems were FGC-related. Recent results [54] illustrating conflicting opinions and approaches concerning menstrual pain and FGC among Swedish healthcare providers support this latter finding. Such conflicting messages hindered most participants from identifying their candidacy for the FGC-specialized services. This finding highlights the difficulty facing girls and women with FGC in identifying health problems as FGC-related, even after receiving relevant information. It also highlights the limitation of focusing on health literacy and exemption from the referral requirement as crucial initiatives for improving access to the FGC-specialized services in Norway. Instead, we think primary healthcare providers with diagnostic competency and FGC knowledge should be responsible for determining the real cause of potential FGC-related problems, particularly as other conditions [18,55,56] could also cause many of these problems.

In the second stage, social networks and some municipalities had helped the participants upon settling in Norway to overcome the language barrier and unfamiliarity with the Norwegian health system, whereas non-profit immigrant organizations were their primary sources of health information on FGC. Former findings [57] have emphasized the role of social networks and immigrant organizations as bridge-builders between immigrants and the healthcare system in Norway. For many years, the Norwegian government has financially supported- and engaged non-profit immigrant organizations in the preventive work on FGC [53]. This preventive framework could be one of the reasons these organizations have focused more on providing information on FGC-related health problems rather than FGC-specialized services as experienced by our study participants. Participants had pondered over lacking knowledge on FGC-competent counselors and claimed it as a reason for not seeking psychosexual counseling. A recent systematic review had also identified the lack of knowledge about FGC-specialized services beyond maternity contexts [58] as a barrier to accessing healthcare in Western countries for women subjected to FGC.

In the third stage, unfamiliarity with FGC-specialized services and uncertainty about FGC as a cause of experienced health problems left the participants un-*ideal users* [47] of these services. Therefore, they chose to book appointments with their regular GPs rather than the FGC-specialized services hoping that the GPs would assess whether their health problems were FGC-related and advise them on the next steps. Nevertheless, feelings of shame, embarrassment, and fear of judgment had discouraged participants from disclosing their FGC status to their GPs and ultimately hindered a proper assessment of FGC as a potential cause for their problems. This finding confirms the hypothesis that ethnic minorities are sensitized to the possibility that some aspects of their cultural practices are prone to judgments [47]. Consistent with a Dutch study [59], our participants had either reluctantly revealed their FGC status while relaying their potential FGC-related health problems or withheld it but expected the GPs to ask them about their FGC status if relevant. When the GPs did not ask them about- or link their health problems to- FGC, participants mostly concluded that the GPs lacked knowledge of FGC or that their health problems were not FGC-related. We do not know the GPs’ exact reasons for not asking about FGC, but former findings [60–62] suggest that perceiving FGC as a taboo and having insufficient knowledge may play a key role. In several Western countries, GPs score low on both the clinical and cultural aspects of FGC [63–65]. In contrast, gynecologists score higher than other healthcare providers [65,66]. Hence, explaining why gynecologists were the ones mostly adjudicating the candidacy to FGC-specialized services among our participants.

In the final stage, sexual norms [44] determined the acceptability of deinfibulation and the participants’ final decision to accept or decline surgery offers. Still, negative dynamics in their interactions with healthcare providers had led some participants to reject deinfibulation offers despite their positive attitudes towards the procedure. The dynamics of interactions with healthcare providers had also influenced participants’ eventual satisfaction with the FGC-specialized services. Participants reported many concerns related to fear of judgment and the process of deinfibulation per se. However, when the healthcare providers met them with respect, provided them with sufficient information about their health problems and deinfibulation, and involved them in the decision-making, the participants felt satisfied and had readily accepted offers of deinfibulation. As patients in Norway are entitled to the information necessary for understanding their condition and treatment options and participate in healthcare decisions [38], it is a surprise that participants had not experienced healthcare providers to accommodate these needs more often. Unfortunately, such negative experiences with healthcare providers also seem widespread among migrants with FGC in other Western countries. In a recent systematic review [58], the authors identified over 30 studies on healthcare experiences where girls and women subjected to FGC had reported feeling exposed and humiliated, judged and stereotyped, and lacking choice, power, and control over their healthcare.

### 4.1 Implication for policy and clinical practice

Our findings suggest that health authorities and healthcare providers in Norway, and possibly other Western countries, need to address several issues to ensure that girls and women subjected to FGC have access to healthcare appropriate to their needs.

Firstly, as girls and women subjected to FGC seem to rely on non-profit immigrant organizations for health information, these organizations could also be encouraged to provide information on FGC-specialized services. Secondly, girls and women subjected to FGC seem to have an unmet need for psychosexual counseling. As a start, psychosexual counseling could be part of the offered FGC-specialized services. Eventually, continuous and comprehensive training of psychosexual counselors on FGC could help to mainstream these services. Thirdly, as GPs are per facto gatekeepers to the FGC-specialized services and whether FGC is the cause for the health problems requires medical knowledge of other possible causes and diagnostic competency, the onus should not be left entirely to the women. Finally, our findings suggest a need for healthcare providers to treat patients, including those subjected to FGC, with respect and compassion, provide sufficient time and information for the patients to make informed decisions, and involve them in the decision-making about their healthcare.

## 5 Conclusion

This article identified a series of barriers and facilitators facing girls and women while accessing FGC-specialized healthcare in Norway.

A key finding is that although girls and women with FGC do not always need referrals to access the Norwegian FGC-specialized healthcare services, none of the participants with potential FGC-related health problems had directly booked appointments with these services. That was mainly due to the participants’ difficulty assessing whether their health problems were FGC-related and unfamiliarity with the FGC-specialized healthcare services. Thus, most participants had first sought medical attention at their GPs. However, when the GPs did not inquire about their FGC status or suggested a link between their health problems and FGC, participants mostly concluded that the GPs lacked knowledge of FGC or that their health problems were not FGC-related. Consequently, the GPs’ inadequate assessment of FGC as a potential cause of health problems delayed the participants’ access to appropriate healthcare. This finding suggests that Norwegian policymakers need to acknowledge the central role of GPs in the clinical management of patients with potential FGC-related health problems and provide them with comprehensive training on FGC. Another key finding is that the interaction dynamics with healthcare providers also influenced the participants’ access to appropriate healthcare. For example, many participants had at one or more points refused offers of deinfibulation because of perceived disrespect, aesthetic concerns, and fear of retraumatization and pain during and after genital surgery. However, positive dynamics where the healthcare providers met the participants with respect and addressed their fears and concerns had facilitated the participants’ acceptance of offers of deinfibulation. Therefore, regardless of their moral stand on FGC, we would urge healthcare providers to meet patients with FGC with respect and consistently provide sufficient information necessary for healthcare decision-making.
